# SRIS: Saliency-Based Region Detection and Image Segmentation of COVID-19 Infected Cases

**DOI:** 10.1109/ACCESS.2020.3032288

**Published:** 2020-10-19

**Authors:** Aditi Joshi, Mohammed Saquib Khan, Shafiullah Soomro, Asim Niaz, Beom Seok Han, Kwang Nam Choi

**Affiliations:** 1 Department of Computer Science and EngineeringChung-Ang University 26729 Seoul 06974 South Korea; 2 Department of Electrical and Electronics EngineeringChung-Ang University 26729 Seoul 06974 South Korea; 3 Quaid-e-Awam University of Engineering, Science and Technology Nawabshah 77150 Pakistan; 4 STARS TeamINRIA 52828 06902 Sophia Antipolis France; 5 School of Food and Pharmaceutical EngineeringHoseo University 34955 Asan 31449 South Korea

**Keywords:** Active contours, image segmentation, level-set

## Abstract

Noise or artifacts in an image, such as shadow artifacts, deteriorate the performance of state-of-the-art models for the segmentation of an image. In this study, a novel saliency-based region detection and image segmentation (SRIS) model is proposed to overcome the problem of image segmentation in the existence of noise and intensity inhomogeneity. Herein, a novel adaptive level-set evolution protocol based on the internal and external functions is designed to eliminate the initialization sensitivity, thereby making the proposed SRIS model robust to contour initialization. In the level-set energy function, an adaptive weight function is formulated to adaptively alter the intensities of the internal and external energy functions based on image information. In addition, the sign of energy function is modulated depending on the internal and external regions to eliminate the effects of noise in an image. Finally, the performance of the proposed SRIS model is illustrated on complex real and synthetic images and compared with that of the previously reported state-of-the-art models. Moreover, statistical analysis has been performed on coronavirus disease (COVID-19) computed tomography images and THUS10000 real image datasets to confirm the superior performance of the SRIS model from the viewpoint of both segmentation accuracy and time efficiency. Results suggest that SRIS is a promising approach for early screening of COVID-19.

## I. Introduction

Numerous image segmentation methods have been proposed for various applications and image types [1]. These methods are broadly categorized as thresholding-based, deformable-based, and deep learning-based [2]. Threshold-based segmentation is the simplest segmentation method, but it requires an optimal threshold value for accurate segmentation. However, the selection of an optimal threshold is very challenging, degrading the performance with improper selection, which further degrades in presence of inhomogeneity and noise. Whereas deep learning models can provide higher accuracy than other methods, they require larger datasets and more computing power [3]. Therefore, the deformable-based active contour model (ACM) has attracted attention over the last two decades.

In the past few years, ACM has achieved the most advanced performance in image processing. From image segmentation and object positioning to object detection and saliency detection, ACM has been extensively used in image analysis, medical imaging, and computer vision tasks [4]. ACM can effectively handle the topological changes of the contour evolution. However, inhomogeneity or pixel variation is a significant problem in image segmentation with ACMs. These problems are caused by defects that occur during image acquisition or by external obstructions. Shadow artifacts that appear on the image degrade the performance of the segmentation methods that assume a constant intensity over the image range. Therefore, inhomogeneity may cause erroneous results and confound the radiologists and experts during diagnosis [5]. Primarily, ACM can be branched as edge-based [6]–[10] and region-based models [11]–[28].

The gradient information of the image is utilized in the edge-based models for contour evolution around the object boundaries. Geodesic ACM (GAC) [7], one of the most prominent edge-based models, integrates edge detectors and gradient information into an evolving curve to construct the edge stopping function. An advantage of this model is that it does not require region-based constraints. Therefore, it can feasibly achieve accurate segmentation results for images with heterogeneous or homogeneous intensities. However, the gradient information is susceptible to noise and largely depends on the contour’s initial position. Therefore, the edge-based models cannot converge to weak edges with noise and low contrast, thereby making it difficult to provide consistent results.

In contrast, region-based models can achieve better results than edge-based models owing to the utilization of the region descriptors for directing the evolving contour movement. The region-based model uses global image information of the internal and external regions for constructing an evolving contour. Therefore, compared with edge-based models, region-based model can accurately obtain segmentation results in the presence of blurred edges. Among the region-based models, the most popular model is Chan–Vese (CV) [12] based on the Mumford–Shah (MS) model [11], which considers the image regions to be homogeneous or constant. In addition, some region-based models override the constraints of the global active contours and consider only the local image information for inhomogeneous image segmentation [13], [15]. However, under extreme noise and specific inhomogeneity conditions, local ACMs are not always suitable to achieve accurate image segmentation. Furthermore, to overcome the problem of inhomogeneity, various bias correction segmentation strategies have been proposed in [14], [18]. In [16], a unique region-based ACM was proposed by integrating a global signed pressure force function, which was characterized using intensity means given in [12].

Recently, several hybrid (local and global) ACMs have been proposed for image segmentation [19]–[25], [27], [28]. For various applications, these models alternatively consolidate region and edge information. For instance, a two-stage hybrid technique was proposed in [22], which coordinates region and edge information in separate phases. In the first phase, the global region and edge information are used to create rough initial segmentation, whereas in the next stage, local pixel and edge information is used to produce the final segmentation results. In [21], [23], a weighted p-Laplace integral that integrated the length regularization term to scale-back the noise effect, while contour minimization was proposed. In addition, [21] has considered a bias field to overcome the inhomogeneities. However, these models are weak in capturing strong intensity inhomogeneity. In [27], hybrid and local fuzzy region-edge based active contour model (HLFRA) is proposed, in which the region energy is strictly convex, and the evolution curve of edge energy stops on the object boundary. In the case of visible object edges, HLFRA performs well in segmenting images with intensity inhomogeneity and noise but fails if the edges are varying.

Saliency has been used in various disciplines, including neurobiology, social neuroscience, image processing, and computer vision. It has been widely implemented in tandem with other approaches for the application of image segmentation [29]–[32]. In image segmentation, saliency refers to the perceptual quality that makes an object, pixel, or person stand out from their neighbors and, thus, attracts our attention. Generally, in the context of visual processing, this technique refers to the unique characteristics (such as pixels or intensity inhomogeneity) of an image. For instance, a color image is converted to a black-and-white image for analyzing the existence of the most intense colors. Therefore, the saliency information can be leveraged for image segmentation. In [29], a saliency-based segmentation method for the color image was proposed, which constructs a facial saliency map and uses it for face segmentation and tracking. In [31], the saliency-SVM (support vector machine) model was proposed, which considered the saliency information and formulated the image segmentation as a binary classification problem. Further, using affinity propagation clustering algorithm, [32] combines regional saliency and uses the random walks method for segmentation. Moreover, visual saliency with ACMs are proposed in [33], [34] to enhance the segmentation results. However, these saliency-based models cannot accurately segment images with weaker edges due to inhomogeneity.

As of August 25, 2020, coronavirus disease 2019, or COVID-19, has spread globally and has caused 23,844,912 confirmed cases and 817,906 mortality cases [35]. The computed tomography (CT) is a non-invasive imaging technique that can detect the characteristics such as ground-glass opacity or bilateral patchy shadows and serves as a practical approach for early screening of COVID-19 [36]. In current medical practice, identification and classification of COVID-19 infection need manual execution, which is a laborious process and requires experienced and well-trained radiologists. To the best of the authors’ knowledge, there has been no attempt to develop an ACM that can segment COVID-19 infection. Segmenting COVID-19 infections from CT images is still a challenging task due to the high variation in size, texture, and position of infections [37]. Therefore, this study aims to develop an ACM that provides efficient and robust segmentation not only on real and synthetic images but also on medical images with intensity inhomogeneity, low contrast, and noise.

The past ACMs are weak to segment real and COVID-19 CT images with severe intensity inhomogeneity and noise, and are sensitive to initialization. Thus, a novel saliency-based region detection and image segmentation (SRIS) model is proposed. Here, a new energy function is designed by incorporating the region saliency and variance of color information in the level-set along with the adaptive weight and sign functions to overcome the issues due to severe intensity inhomogeneity and noise. In addition, a new internal and external energy-based level-set evolution protocol is designed for robust and fast contour evolution.

The major contributions of this study are summarized as follows.

• A novel SRIS model is proposed to overcome the severe inhomogeneity and noise in an image. Herein, a new energy function is derived that can efficiently extract the object with complex background regardless of severe inhomogeneity and noise by incorporating the saliency and variance of color information in the level-set.
• A new level-set evolution protocol is designed based on the internal and external energy functions. The aim is to ensure that the proposed SRIS model is robust to initialization and converges significantly faster than the other segmentation models.
• Adaptive weight and sign functions are formulated in the energy function to accomplish inhomogeneous image segmentation and robustness to noise, respectively.
• After the implementation of the proposed SRIS model, the contour evolutions over numerous real and synthetic images are performed to confirm the efficiency and superiority of the proposed SRIS model than the state-of-the-art models.

The remainder of the paper is structured as follows. Related work is described in Section II. In Section III, the proposed SRIS model is derived using the information on saliency and variance of color intensity. Precisely, the mathematical implementation of the proposed energy function for contour evolution is developed. In Section IV, the simulation results show the performance of the proposed SRIS model on synthetic and real images and the results are compared with those of the state-of-the-art models. Quantitative and qualitative analyses for the state-of-the-art and proposed models are performed in Section V using COVID-CT and THUS10000 real image datasets. Finally, the conclusions are drawn in Section VI.

## II. Related Work

This section presents state-of-the-art ACMs: CV [12], variational level-set for bias correction and segmentation (VLSBCS) [14], local statistical ACM (LSACM) [18], local and global fitted image (LGFI) [19], and fuzzy region-based active contours driven by weighting global and local fitting energy (FRAGL) [24]. Subsequently, in Sections IV and V, these models are compared with the proposed SRIS model.

### A. CV Model

In [12], the CV model was proposed, which is an ACM based on the MS model [11] for the global image segmentation. Let $I:\Omega \to {\Re ^{2}}$ be an input image, $\Omega $ be an image domain, $\Phi:\Omega \to {\Re ^{2}}$ be a level-set function, and Contour $C:\left \{{ {x \in \Omega \left |{ {\phi \left ({x }\right) = 0} }\right.} }\right \}$ be the zero level-set. The energy function of the CV model is defined as

\begin{align*}&\hspace {-2pc}E_{\text {CV}}\left ({{C,{c_{1}},{c_{2}}} }\right) \\[2pt]=&{\lambda _{1}}\int \limits _\Omega {{{\left |{ {I\left ({x }\right) - {c_{1}}} }\right |}^{2}}{H_\varepsilon }\left ({{\phi \left ({x }\right)} }\right)dx} \\[2pt]&+\, {\lambda _{2}}\int \limits _\Omega {{{\left |{ {I\left ({x }\right) - {c_{2}}} }\right |}^{2}}\left [{ {1 - {H_\varepsilon }\left ({{\phi \left ({x }\right)} }\right)} }\right]dx} \\[2pt]&+\, \mu \int \limits _\Omega {{{\left |{ {\nabla {H_\varepsilon }\left ({{\phi \left ({x }\right)} }\right)} }\right |}^{2}}dx} + \nu \int \limits _\Omega {H_\varepsilon \left ({{\phi \left ({x }\right)} }\right)dx}, \tag{1}\end{align*}

where $\left \{{ {\mu,v,{\lambda _{1}},{\lambda _{2}}} }\right \} \ge 0$ are constant positive parameters and {$c_{1}$, $c_{2}$} denote mean image intensity {inside, outside} contour $C$. Herein, $\mu $ and $v$ control the length term and area term for contour $C$, respectively. In addition, the smooth approximated Heaviside function ${H_\varepsilon }\left ({\phi }\right)$, where $\varepsilon $ balances the smoothness is given as

\begin{equation*} {H_\varepsilon }\left ({\phi }\right) = \frac {1}{2}\left [{ {1 + \left ({{\frac {2}{\pi }} }\right)\arctan \left ({{\frac {\phi }{\varepsilon }} }\right)} }\right] \tag{2}\end{equation*}

As a global ACM, the CV model’s contour evolution is analogous to the global characteristics of an image region. Consequently, it weakens with images that have local or inhomogeneous-intensity regions.

### B. VLSBCS Model

In [14], a VLSBCS model has been proposed for bias correction and segmentation for images with intensity inhomogeneities. Herein, the proposed energy function ensures that the evaluated bias field is smooth, without any additional computation to maintain the smoothness of the bias field. The VLSBCS is based on a model that commonly describes images with inhomogeneous intensity as

\begin{equation*} I\left ({x }\right) = b\left ({x }\right)J\left ({x }\right) + n\left ({x }\right), \tag{3}\end{equation*}

where $I\left ({x }\right)$, $b\left ({x }\right)$, $J\left ({x }\right)$, and $n\left ({x }\right)$ denote the input image with intensity inhomogeneity, bias field responsible for intensity inhomogeneity, original image, and noise, respectively. In (3), it is assumed that $b\left ({x }\right)$ varies slowly throughout the image domain, and $J\left ({x }\right) \approx {c_{i}}$ for $x \in {\Omega _{i}}$, with $x \in \left ({{\Omega _{i}} }\right)_{i = 1}^{N}$, is approximately a constant $c_{i}$ within each object in the image, where $N$ is a number of disjoint regions or clusters.

This model used K-means clustering for local image intensities classification and formulated an energy function

\begin{align*} E_{\text {VLSBCS}} \stackrel { \Delta } = \int {\left ({\! {\sum \limits _{i = 1}^{N} {\int \limits _{\Omega _{i}} {\kappa _\rho \left ({{x - y} }\right){{\left |{ {I\left ({y }\right) - b\left ({x }\right){c_{i}}} }\right |}^{2}}dy} } } \!}\right)dx}. \\\tag{4}\end{align*}

In the case of $N=2$, image domain $\left \{{ {\Omega _{i}} }\right \}_{i = 1}^{N}$ can be partitioned into two regions, $\left \{{ {\Omega _{i}} }\right \}_{i = 1}^{2}$, separated by zero level-sets such that ${\Omega _{1}} \cong \phi > 0$ and ${\Omega _{2}} \cong \phi < 0$. Then, (4) becomes

\begin{align*}&\hspace {-1.2pc}E_{\text {VLSBCS}}\left ({{\phi,b,{\mathbf {c}}} }\right) \\=&\int \!\! {\left ({\! {\sum \limits _{i = 1}^{2} {\int {\kappa _\rho \left ({{x \!- \!y} }\right){{\left |{ {I\left ({y }\right) \!- \!b\left ({x }\right){c_{i}}} }\right |}^{2}}} {M_{i}}\left ({\phi }\right)dy} } \!}\right)dx}, \tag{5}\end{align*}

where ${M_{1}} = {\mathrm{ H_\varepsilon }}\left ({\phi }\right)$ and ${M_{2}} = \left ({{1 - {\mathrm{ H_\varepsilon }}\left ({\phi }\right)} }\right)$. This model is robust on intensity inhomogeneity regions only when edges are visible (even if the edges are blurred). In addition, VLSBCS is dependent on initial contour’s position and fails in severe intensity inhomogeneity.

### C. LSACM Model

In [18], the LSACM model has been proposed. It corrects and segments the bias field in an intensity inhomogeneous image. Herein, an image with intensity inhomogeneity was represented as Gaussian distributions with different means and variances. In addition, they are projected with the multiplication of a bias field with the real image in a Gaussian window. An energy function of LSACM was given as

\begin{align*}&\hspace {-.5pc} E_{\text {LSACM}} =\!\sum \limits _{i = 1}^{N}\! {\int \limits _\Omega {\kappa _\rho \left ({{x \!- \!y} }\right){M_{i}}\left ({\phi }\right)}} \\&\qquad\qquad\qquad\quad \displaystyle {{{\times \,\left ({\! {\log \left ({{\sigma _{i}} }\right) \!+ \! \frac {{{{\left ({{I\left ({y }\right) \!- \!b\left ({x }\right){c_{i}}} }\right)}^{2}}}}{2\sigma _{i}^{2}}} \!}\right)dx} }, } \tag{6}\end{align*}

where ${\sigma _{i}}$ denotes the variances:

\begin{equation*} {\sigma _{i}} \!=\! \sqrt {\frac {{\int {\int {\kappa _\rho \left ({{x \!- \!y} }\right){M_{i}}\left ({{\phi \left ({y }\right)} }\right){{\left ({{I\left ({y }\right) - b\left ({x }\right){c_{i}}} }\right)}^{2}}dy} dx} }}{{\int {\int {\kappa _\rho \left ({{x - y} }\right){M_{i}}\left ({{\phi \left ({y }\right)} }\right)dy} dx} }}}. \tag{7}\end{equation*}

This model is very effective for image segmentation with inhomogeneity but fails to find the precise image boundary.

### D. LGFI Model

In [19], an ACM has been proposed, which targeted intensity inhomogeneity on LGFI. The model’s energy function was given as

\begin{equation*} E_{\text {LGFI}} = \int \limits _\Omega {\left ({{I\left ({x }\right) - {I_{bLFI}}\left ({x }\right)} }\right)} \left ({{I\left ({x }\right) - {I_{GFI}}\left ({x }\right)} }\right)dx, \tag{8}\end{equation*}

where ${I_{bLFI}}$ and ${I_{GFI}}$ are the bias local fitted image and the global fitted image:

\begin{align*} {I_{bLFI}}\left ({x }\right)=&b\left ({x }\right)\left ({{c_{1}{M_{1}} + {c_{2}}{M_{2}}} }\right), \tag{9}\\ {I_{GFI}}\left ({x }\right)=&{f_{1}}{M_{1}} + {f_{2}}{M_{2}}, \tag{10}\end{align*}

where $f_{1}$, $f_{2}$ and $c_{1}$, $c_{2}$ are global and local mean intensities, respectively. In this model, a bias field is considered only for the local contour evolution and not for the global one. Thus, it can only solve the problem of local intensity inhomogeneity segmentation.

### E. FRAGL Model

In [24], FRAGL ACM was proposed, which integrated the fuzzy sets to achieve a convex global energy function. The FRAGL model is different from other ACMs, as it uses 0.5 as the level-set for the contour evolution. This pseudo level-set function, $u(x)$, was given as

\begin{align*} \begin{cases} {u\left ({x }\right) = 0.5},&{I\left ({x }\right) \in C},\\ {u\left ({x }\right) > 0.5},&{I\left ({x }\right) \in In\left ({C }\right)},\\ {u\left ({x }\right) < 0.5},&{I\left ({x }\right) \in Out\left ({C }\right)}, \end{cases} \tag{11}\end{align*}

where $Out\left ({C }\right)$ and $In\left ({C }\right)$ denote the region outside and inside contour $C$. The energy function of FRAGL was

\begin{equation*} E_{\text {FRAGL}}\left ({u }\right) = {l_{1}}L\left ({{u - 0.5} }\right) + {l_{2}}P\left ({{u - 0.5} }\right), \tag{12}\end{equation*}

where $L$, $P$, and $l_{1}$, $l_{2}$ denote a regularization term, penalty term, and positive parameters, respectively. $L$ helps to evolve the length of the contour to ensure that the pseudo level-set function remains smooth:

\begin{equation*} L\left ({{u = 0.5} }\right) = \int \limits _\Omega \delta \left ({{u - 0.5} }\right)\left |{ {\nabla \left ({{u - 0.5} }\right)} }\right |dx. \tag{13}\end{equation*}

Moreover, $P$ keeps the consistency within the signed distance function and pseudo level-set function:

\begin{equation*} P\left ({{u = 0.5} }\right) = \frac {1}{2}{\int \limits _\Omega {\left ({{1 - \left |{ {\nabla \left ({{u - 0.5} }\right)} }\right |} }\right)} ^{2}}dx, \tag{14}\end{equation*}

where $\delta = {\varepsilon \mathord {\left /{ {\vphantom {\varepsilon {\pi \left ({{\phi ^{2} + {\varepsilon ^{2}}} }\right)}}} }\right. } {\pi \left ({{\phi ^{2} + {\varepsilon ^{2}}} }\right)}}$ and $\nabla $ denote Dirac delta function and Hamilton operator, respectively.

## III. Proposed SRIS Model

In this section, a new SRIS model is proposed to overcome the problem with severe intensity inhomogeneity and noise in an image. Herein, a new energy function is derived that can efficiently extract the object with complex background regardless of severe inhomogeneity and noise. In addition, a new level-set evolution protocol is designed based on internal and external energy functions such that the proposed SRIS model is robust to initialization and converges significantly faster than other segmentation models.

Let $I:\Omega \to {\Re ^{2}}$ be an input image and $\phi $ be the level-set function with the initial contour $C:\{x \in \Omega |\phi = 0\}$ in an image domain $\Omega $. In addition, let $\Omega _{0}:\{\Omega |\phi = 0 \}$ be the zero level-set, and $\Omega _{\text {in}}:\{\Omega |\phi < 0 \}$ and $\Omega _{\text {ex}}:\{\Omega |\phi > 0 \}$ be a domain inside and outside ${\Omega _{0}}$, respectively. The proposed energy function $E_{\text {SRIS}}$ is defined as

\begin{equation*} E_{\text {SRIS}} = E_{\text {ex}}\left ({{\phi } }\right) + E_{\text {in}}\left ({{\phi } }\right). \tag{15}\end{equation*}

Herein, the external energy function $E_{\text {ex}}$ is determined by region, gradient, and saliency, while the internal energy function $E_{\text {in}}$ is used as a constraint for the evolution of level-set.

In an image with intensity inhomogeneity and color variation, the pixels are clustered and the pixels with similar intensity and saliency values are assigned to both $\Omega _{\text {in}}$ and $\Omega _{\text {ex}}$. Thus, the proposed external energy function $E_{\text {ex}}$ incorporates the saliency information as well as the variance of color intensity for both $\Omega _{\text {in}}$ and $\Omega _{\text {ex}}$ of the input image, $I$.

\begin{align*}&\hspace {-1.2pc}E_{\text {ex}}\left ({\phi }\right) \\=&\alpha \left [{ {\int \limits _\Omega {h{Y_{\text {in}}(x)}{H_\varepsilon }\left ({\phi }\right)} dx + \int \limits _\Omega {h{Y_{\text {ex}}(x)}\left ({{1 - {H_\varepsilon }\left ({\phi }\right)} }\right)} dx} }\right] \\&+\, \lambda \left [{ {\int \limits _\Omega {h{Z_{\text {in}}}(x)} {H_\varepsilon }\left ({\phi }\right)dx \!+ \!\int \limits _\Omega {h{Z_{\text {ex}}}(x)} \left ({{1 - {H_\varepsilon }\left ({\phi }\right)} }\right)dx} \!}\right]\!, \\ \tag{16}\end{align*}

where

\begin{align*} {Y_{\text {in}}(x)}=&{\left [{ {S(x) - {s_{1}}} }\right]^{2}},\,{Y_{\text {ex}}(x)} = {\left [{ {S(x) - {s_{2}}} }\right]^{2}}, \tag{17}\\ {Z_{\text {in}}}\left ({x }\right)=&{\left |{ {I\left ({x }\right) - {c_{1}}} }\right |^{2}} + {\left |{ {I\left ({x }\right) - f} }\right |^{2}}, \\ {Z_{\text {ex}}}\left ({x }\right)=&{\left |{ {I\left ({x }\right) - {c_{2}}} }\right |^{2}}, \tag{18}\end{align*}

$\varepsilon \ge 0$, ${\mathrm{ H_\varepsilon }}\left ({\phi }\right)$ is $\varepsilon -$Heaviside function given in (2), and $\{\alpha, \lambda \} > 0 $ are fixed scaling constants for saliency information (first term) and variance of color intensity (second term), respectively. In addition, both saliency information and variance of color intensity energy functions in (16) are embedded with an edge indicator, $h$, described as

\begin{equation*} h = \frac {1}{{1 + {{\left |{ {\nabla {\kappa _\rho }*I} }\right |}^{2}}}}, \tag{19}\end{equation*}

where $\nabla $ and ${\kappa _\rho }$ are the gradient operator and Gaussian kernel with standard deviation $\rho $. In addition, * is the convolution operator that reduces the influence of intense noise.

In (17), $S$ is the saliency information, which aims at identifying the most distinct objects or regions such as edge, color, and/or texture in an image. It is formulated as [38]:

\begin{equation*} S(x) = \left |{ {\bar I(x) - {I_\kappa }(x)} }\right |, \tag{20}\end{equation*}

where $\bar I$ is the mean pixel value of $I$ and $I_\kappa = {\kappa _\rho } * I$ is the image blurred by the Gaussian filter. Moreover, $s_{1}$ and $s_{2}$ are the saliency means for $\Omega _{\text {in}}$ and $\Omega _{\text {ex}}$, respectively:

\begin{align*} {s_{1}} = \frac {{\int \limits _\Omega {S(x) \cdot {H_\varepsilon }\left ({\phi }\right)dx} }}{{\int \limits _\Omega {H_\varepsilon \left ({\phi }\right)dx} }},\quad {s_{2}} = \frac {{\int \limits _\Omega {S(x) \cdot \left ({{1 - {H_\varepsilon }\left ({\phi }\right)} }\right)dx} }}{{\int \limits _\Omega {\left ({{1 - {H_\varepsilon }\left ({\phi }\right)} }\right)dx} }}. \\ \tag{21}\end{align*}

In (18), $c_{1}$ and $c_{2}$ are the scalar approximation of the mean intensities for $\Omega _{\text {in}}$ and $\Omega _{\text {ex}}$, respectively, and $f$ is the scalar approximation of the median for $\Omega _{\text {in}}$.

\begin{align*} {c_{1}}=&\omega \cdot {\text {mean}}\left ({{I(x) \in {\Omega _{\text {in}}}} }\right), \\ {c_{2}}=&\omega \cdot {\text {mean}}\left ({{I(x) \in {\Omega _{\text {ex}}}} }\right), \\ f=&\omega \cdot {\text {median}}\left ({{I(x) \in {\Omega _{\text {in}}}} }\right), \tag{22}\end{align*}

where $\omega $ is an adaptive weighted function:

\begin{align*}&\hspace {-.5pc} \omega = \int _\Omega {H_\varepsilon \left ({\phi }\right){{\left \|{ {{Z_{\text {in}}}(x)} }\right \|}_{2}}dx} \\&\qquad\qquad\qquad\quad\; \displaystyle { +\, \int _\Omega {\left ({{1 - {H_\varepsilon }\left ({\phi }\right)} }\right){{\left \|{ {{Z_{\text {ex}}}(x)} }\right \|}_{2}}dx}, } \tag{23}\end{align*}

where $\left \|{ \cdot }\right \|$ is the L2 norm. Compared to the mean values, the median is closer to the pixel value of the object boundary, which can effectively suppress the noise and retain more detailed features such as thin lines.

However, only with the external energy function, the segmentation may be inaccurate and irregular, and some singularities or undesired false contour may appear. Therefore, the internal energy function is given as

\begin{equation*} E_{\text {in}}\left ({\phi }\right) = \frac {\mu \ell \left ({\phi }\right)\eta \left ({I }\right)}{{\max \left ({{\left |{ {\eta \left ({I }\right)} }\right |} }\right)}} + \nu {P}\left ({\phi }\right), \tag{24}\end{equation*}

where $\mu, \nu > 0$ are constants. Terms ${\ell }\left ({\phi }\right)$ and ${P}\left ({\phi }\right)$ are the weighted length term of the contour dealing with object’s boundary based on edge information

\begin{equation*} {\ell }\left ({\phi }\right) = \int \limits _\Omega {h\delta _\varepsilon \left ({\phi }\right)\left |{ {\nabla \phi } }\right |dx}, \tag{25}\end{equation*}

and area term of the contour to calculate the region-of-interest (ROI)

\begin{equation*} {P}\left ({\phi }\right) = \int \limits _\Omega {\frac {1}{2}{{\left ({{1 - \left |{ {\nabla \phi } }\right |} }\right)}^{2}}dx}, \tag{26}\end{equation*}

respectively. Here, ${\delta _\varepsilon } = {\varepsilon \mathord {\left /{ {\vphantom {\varepsilon {\pi \left ({{\phi ^{2} + {\varepsilon ^{2}}} }\right)}}} }\right. } {\pi \left ({{\phi ^{2} + {\varepsilon ^{2}}} }\right)}}$ is a Dirac delta function. ${E_{\text {in}}}$ is to regularize $\phi $ with the use of $P (\phi)$ such that the contour remain close to the ROI and prevent the appearance of singularity for smooth contour evolution. Term $\eta \left ({I }\right)$ is to modulate the signs of the length term using the statistics defined in (22):

\begin{equation*} \eta (I)\,\,= {\text {sgn}}\left ({{2{c_{1}} + 2f - 4{c_{2}}} }\right) \cdot {\text {sgn}}\left ({\Upsilon }\right) \cdot {\Upsilon ^{2}}, \tag{27}\end{equation*}

where

\begin{equation*} \Upsilon = {I(x) - \frac {{c_{1}^{2} + {f^{2}} - 2c_{2}^{2}}}{{2{c_{1}} + 2f - 4{c_{2}}}}}, \tag{28}\end{equation*}

and $\mathrm {sgn} \left ({\cdot }\right)$ is the signum function with the values {−1, 1} for $\Omega _{\text {in}}$ and $\Omega _{\text {ex}}$ domains of $I$.

Hence, the proposed energy function $E_{\text {SRIS}}$ in (15) can be rewritten as

\begin{align*}&\hspace {-1.2pc}E_{\text {SRIS}}\left ({\phi }\right) \\[3pt]=&\alpha \left [{ {\int \limits _\Omega {h{Y_{\text {in}}(x)}{H_\varepsilon }\left ({\phi }\right)dx} + \int \limits _\Omega {h{Y_{\text {ex}}(x)}\left ({{1 - {H_\varepsilon }\left ({\phi }\right)} }\right)dx} } }\right] \\[3pt]&+\, \lambda \left [{\! {\int \limits _\Omega {h{Z_{\text {in}}(x)}{H_\varepsilon }\left ({\phi }\right)dx} \!+ \!\int \limits _\Omega {h{Z_{\text {ex}}(x)}\left ({{1 - {H_\varepsilon }\left ({\phi }\right)} }\right)dx} } \!}\right] \\[3pt]&+\, \frac {\mu \eta \left ({I }\right)}{{\max \left ({{\left |{ {\eta \left ({I }\right)} }\right |} }\right)}}\int \limits _\Omega {h{\delta _\varepsilon }\left ({\phi }\right)\left |{ {\nabla \phi } }\right |dx} \\[3pt]&+\, \frac {\nu }{2}\int \limits _\Omega {{{\left ({{1 - \left |{ {\nabla \phi } }\right |} }\right)}^{2}}dx}. \tag{29}\end{align*}

Further, to minimize (29) with respect to $\phi $, the derivative of (29) can be written by the calculus of variations

\begin{align*} \frac {{\partial E_{\text {SRIS}}}}{\partial \phi }=&\alpha \left [{ {h{{\left ({{S\left ({x }\right) - {s_{1}}} }\right)}^{2}} - h{{\left ({{S(x) - {s_{2}}} }\right)}^{2}}} }\right] \\&+\, \lambda \left [{\! {h\left ({{{{\left |{ {I(x) \!-\! {c_{1}}} }\right |}^{2}} \!+\! {{\left |{ {I(x) - f} }\right |}^{2}}} }\right) \!- \!h{{\left |{ {I(x) \!-\! {c_{2}}} }\right |}^{2}}} \!}\right] \\&-\, \frac {{\mu \eta \left ({I }\right)\left |{ {\nabla \phi } }\right |}}{{\max \left ({\! {\left |{ {\eta \left ({I }\right)} }\right |} \!}\right)}}{\delta _\varepsilon }\left ({\phi }\right)\text {div}\left ({{h\frac {\nabla \phi }{{\left |{ {\nabla \phi } }\right |}}} }\right)\! - \!\nu \Delta \phi, \tag{30}\end{align*}

where $\text {div}(\cdot)$ and $\Delta $ denote divergence and Laplacian operators, respectively. Using the steepest gradient descent [39] such that $\partial {E_{\text {SRIS}}}/\partial \phi = 0$ (Euler–Lagrange equation), the evolution of $\phi $ in (15) with time $t$ is

\begin{align*} \frac {\partial \phi }{\partial t}=&- \frac {{\partial {E_{\text {SRIS}}}}}{\partial t} \\=&- \alpha h\left [{ {{Y_{\text {in}}(x)} - {Y_{\text {ex}}(x)}} }\right] - \lambda h\left [{ {{Z_{\text {in}}(x)} - {Z_{\text {ex}}(x)}} }\right] \\&+\, \frac {{\mu \eta \left ({I }\right)\left |{ {\nabla \phi } }\right |}}{{\max \left ({{\left |{ {\eta \left ({I }\right)} }\right |} }\right)}}{\delta _\varepsilon }\left ({\phi }\right)\text {div}\left ({{h\frac {\nabla \phi }{{\left |{ {\nabla \phi } }\right |}}} }\right) + \nu \Delta \phi. \tag{31}\end{align*}

In the proposed SRIS model, the region information (such as saliency and variance of color intensity) is a global feature determined by $I$. Therefore, the initialization of $\phi $ can be very flexible. The proposed SRIS’s level-set function is initialized as

\begin{equation*} {\phi _{t = 0}} = \begin{array}{cccccccccccccccccccc} p,&\quad {x \in \Omega, } \end{array} \tag{32}\end{equation*}

where $p \ge 0$ is a constant initial level-set parameter. The evolution of $\phi $ should be stopped using threshold $\gamma $ as follows:

\begin{equation*} \left |{ {\frac {\partial \phi }{\partial t}} \Delta t}\right | = \left |{ {{\phi _{t + 1}} - {\phi _{t}}} }\right | < \gamma, \tag{33}\end{equation*}

because $\phi $ will not converge anymore. Herein, $\Delta t$ is the initial parameter time step. Finally, the proposed SRIS model is summarized in **Algorithm 1**.

Algorithm 1 **Proposed SRIS Model**
Input: $I$, $\lambda $, $\alpha $, $\mu $, $\nu $, and $\gamma $
Output: ${\phi }$
1: **Initialization:** ${\phi _{0}}$ by (32) and $\omega = 1$
2: **for** 1 to maximum iteration **do**
3: Compute edge detector $h$ by (19)
4: Compute saliency information $S(x)$ by (20)
5: Compute saliency means $s_{1}$ and $s_{2}$ by (21) and intensity means $c_{1}$, $c_{2}$, and median $f$ by (22)
6: Update $\omega $ by (23)
7: Compute $\eta (I)$ by (27)
8: Perform level-set evolution by (31)
9: **if** (33) is satisfied **then**
10: Jump to step 3
11: **else**
12: Stop evolution
13: **end if**
1: **end for**

## IV. Simulations and Results

The proposed SRIS model was implemented in MATLAB running on a 3.60 GHz Intel Core i7 system with 8 GB RAM. Multiple synthetic and real images were tested with the proposed SRIS model and compared with the state-of-the-art models such as CV, VLSBCS, LSACM, LGFI, FRAGL (Section II), and HLFRA. The parameters of the proposed SRIS model were fixed throughout the experiments and are given in Table 1, while the parameters of the past models were selected from their respective work.

**TABLE 1:** Parameters of the Proposed SRIS Model

Parameter	Symbol	Value
Weight of color intensity energy	$\lambda $	0.01
Weight of saliency energy	$\alpha $	0.06
Penalization constant of the length of contour	$\mu $	5
Regularization constant of the area of contour	$\nu $	0.2
Constant for Heaviside and Dirac delta functions	$\varepsilon $	1.5
Standard deviation of Gaussian distribution	$\rho $	0.8
Threshold	$\gamma $	$N \times 0.005$
Time step	$\Delta t$	1

*$N$ denotes the number of pixels in $I$.

**FIGURE 1.**
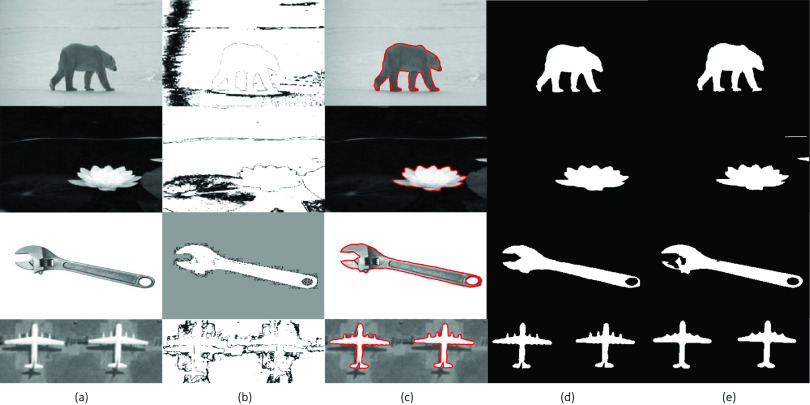
Example of segmentation with and without saliency information. (a) Original image, (b) saliency information, (c) final contour with saliency, (d) result with saliency, and (e) result without saliency.

Fig. 1 demonstrates the comparative segmentation results of the SRIS model with and without saliency information incorporated in the proposed level-set. Herein, two synthetic and two real images (Fig. 1(a)) are used as an input image. The saliency information obtained using the proposed model is shown in Fig. 1(b). The final contour and segmentation results obtained using the proposed SRIS model (i.e., with saliency information) are shown in Fig. 1(c) and Fig. 1(d). Further, to obtain the segmentation result without saliency information, the scale factor of the saliency term $\alpha $ is initialized to zero, and the result is shown in Fig. 1(e). The figure shows that by incorporating the saliency information, the proposed model provides more reliable and accurate segmentation results.

**FIGURE 2.**
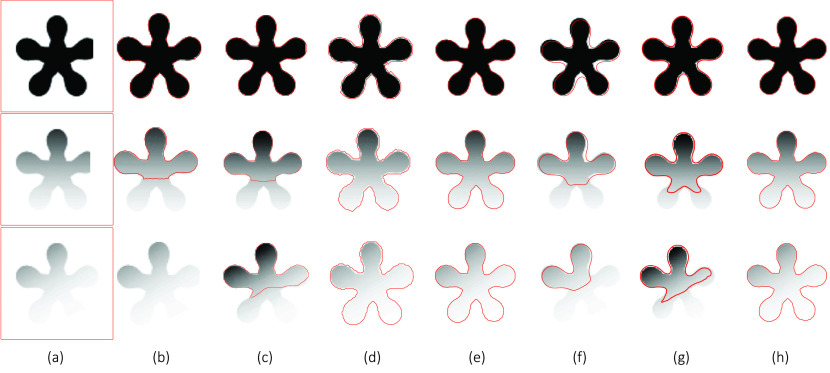
Comparison of segmentation results of a synthetic image with varying intensity. (a) Original image with initial Contour, (b) CV, (c) VLSBCS, (d) LSACM, (e) LGFI, (f) FRAGL, (g) HLFRA, and (h) proposed SRIS.

Fig. 2 compares the segmentation results of a synthetic image with varying intensity obtained with all the methods summarized in Section II and the proposed SRIS model. Here, a homogeneous image (Fig. 2a: Row 1) was used, in which the distribution of intensity has altered to a certain extent (Fig. 2a: Rows 2 and 3), where even manual segmentation becomes difficult to segment the inhomogeneous image well. In Fig. 2, the number of input images with initial contours are shown in Fig. 2(a) followed by the segmentation results obtained by CV, VLSBCS, LGFI, FRAGL, HLFRA, and proposed SRIS models, respectively. As can be seen from the results in Fig. 2, the proposed SRIS model and LGFI model provided the best segmentation results regardless of intensity inhomogeneity. Segmentation results using LSACM showed that it could not find the precise object boundary. In addition, the CV, VLSBCS, and HLFRA models provided close segmentation of the homogeneous (Row 1) image; however, the segmentation performance deteriorated as the intensity inhomogeneity was increased. The segmentation results with the FRAGL model indicated that even for the homogeneous image, it could not find the precise object boundaries.

**FIGURE 3.**
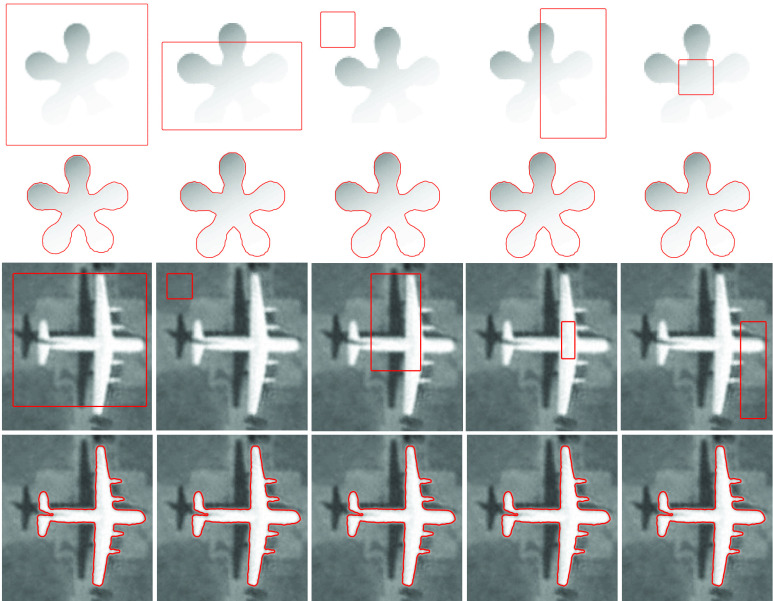
Effect of initial contour position on proposed SRIS model. Rows 1 and 3: Original images with initial contours and Rows 2 and 4: Segmentation results.

In general, most state-of-the-art ACMs need to initialize the level-set function and are considerably sensitive to the initial contour position. However, the proposed SRIS model is robust to the initial contour position, and it provided identical results regardless of the contour position. Fig. 3 shows the robustness toward the initial contour position on two severe intensity inhomogeneous synthetic images. The first and third rows in Fig. 3 show initial contours with different positions to confirm the independence and stability of the proposed SRIS model. The second and fourth rows in Fig. 3 show the final segmentation results relative to different initial contour positions. Even if the edges of the object are blurred and/or invisible, the proposed model is robust to the initial contour position and achieved accurate segmentation regardless of severe intensity inhomogeneity. Therefore, in this article, a constant initial level-set function, $p=1$, is used for all the images.

**FIGURE 4.**
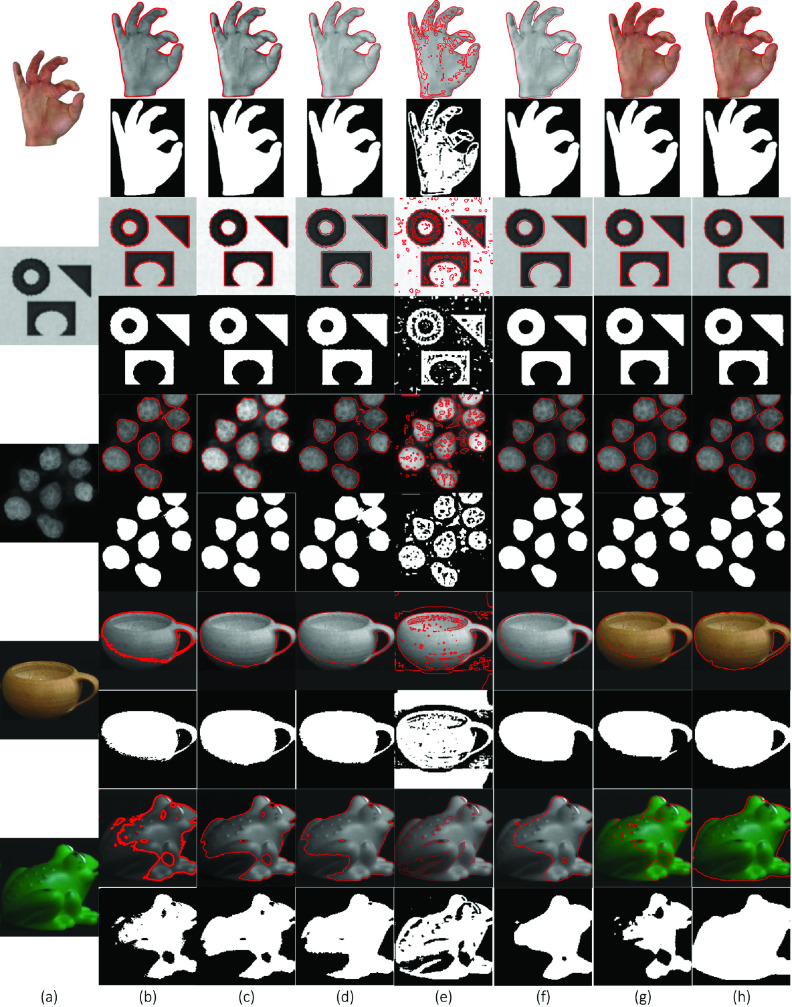
Segmentation results and comparison of synthetic images. (a) Original images, (b) CV, (c) VLSBCS, (d) LSACM, (e) LGFI, (f) FRAGL, (g) HLFRA, and (h) Proposed SRIS.

Fig. 4 illustrates the performance of the proposed model compared to the state-of-the-art models with homogeneous and intensity inhomogeneous synthetic images. The original images are shown in Fig. 4(a), and the subsequent columns show the segmentation results obtained by the CV, VLSBCS, LSACM, LGFI, FRAGL, HLFRA, and proposed SRIS models, respectively. Fig. 4(b) shows the segmentation results of the CV model: images with homogeneous backgrounds were segmented accurately, but if the image contains inhomogeneities in the background and/or foreground, the model cannot accurately capture the ROI. The segmentation results obtained by the VLSBCS, LSACM, FRAGL, and HLFRA models (Fig. 4 (c), (d), (f), and (g)) achieved accurate results on images with homogeneous and inhomogeneous backgrounds but failed to segment the ROI of the last two images with the inhomogeneous foregrounds. The LGFI model cannot obtain the desirable segmentation results, as shown in Fig. 4(e). According to Fig. 4, the segmentation results of the proposed SRIS model (Fig. 4(h)) provided the best results on both types of images, and it accurately extracted the ROI from the foreground.

To evaluate the computational efficiency of the proposed SRIS and state-of-the-art models, the number of iterations required for the contour convergence and final convergence time (i.e., processing time) of all the synthetic images in Fig. 4 are shown in Table 2. The proposed SRIS model can segment the synthetic image with a significantly fewer number of iterations and less processing time compared with the state-of-the-art models. The computational efficiency of FRAGL and HLFRA are close to the proposed SRIS model, but they cannot obtain accurate segmentation of all synthetic images.

**TABLE 2:** Number of Iterations and Processing Time for Fig. 4

Model		Image 1	Image 2	Image 3	Image 4	Image 5
CV	Iteration	200	200	200	500	80
	Processing time (s)	2.39	3.425	2.427	9.429	2.077
VLSBCS	Iteration	50	50	50	10	10
	Processing time (s)	6.756	12.727	5.15	1.99	1.359
LSACM	Iteration	20	20	20	20	40
	Processing time (s)	34.766	153.685	32.61	257.056	78.769
LGFI	Iteration	100	50	100	180	100
	Processing time (s)	5.051	2.899	4.826	9.184	5.437
FRAGL	Iteration	20	30	20	15	20
	Processing time (s)	0.707	1.203	0.6899	0.807	0.799
HLFRA	Iteration	5	5	15	25	25
	Processing time (s)	0.299	0.343	1.348	1.19	1.268
Proposed SRIS	Iteration	5	5	15	10	12
	Processing time (s)	0.286	0.316	0.674	0.429	0.69

**FIGURE 5.**
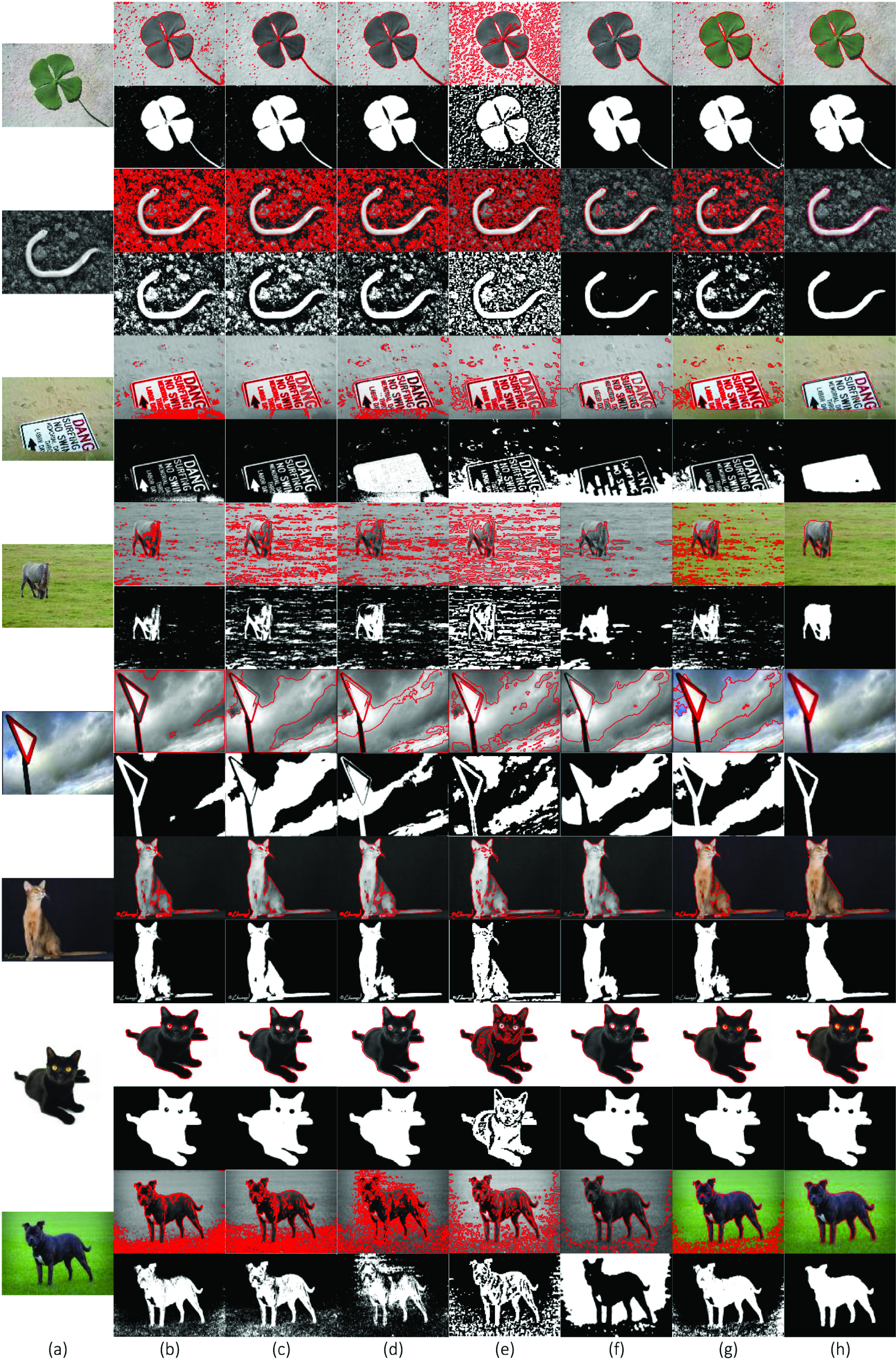
Segmentation and comparison of real images. (a) Original images, (b) CV, (c) VLSBCS, (d) LSACM, (e) LGFI, (f) FRAGL, (g) HLFRA, and (h) Proposed SRIS.

Fig. 5 shows the segmentation results on eight publicly available real images with challenging foregrounds and backgrounds. The original images are shown in Fig. 5(a), and the results are shown in Fig. 5 (b) CV, (c) VLSBCS, (d) LSACM, (e) LGFI, (f) FRAGL, (g) HLFRA, and (h) Proposed SRIS. According to the results, all the state-of-the-art models cannot segment real images with complex backgrounds, except for the seventh image, which is composed of homogenous foreground and background. In addition, the FRAGL model provided near-accurate segmentation of first, second, third, and seventh images (Fig. 5(f)) but failed to segment other images with severe intensity inhomogeneity in the foreground and background. The proposed SRIS model has provided accurate segmentation and precisely extracted the ROI even from highly complex background images.

By analogy with Table 2, Table 3 shows the performance of the proposed SRIS model in terms of the number of iterations and processing time required for all the real images in Fig. 5 compared with the previous models. The proposed SRIS model consumed relatively fewer iterations and less processing time than previous models.

**TABLE 3:** Number of Iterations and Processing Time for Fig. 5

Model		Image 1	Image 2	Image 3	Image 4	Image 5	Image 6	Image 7	Image 8
CV	Iteration	80	80	800	200	20	200	100	100
	Processing time (s)	2.985	2.923	18.372	20.164	1.94	12.64	3.568	6.503
VLSBCS	Iteration	40	40	40	20	20	20	20	20
	Processing time (s)	18.039	17.219	17.8	28.133	13.949	18.989	6.529	16.226
LSACM	Iteration	40	40	20	20	20	20	20	20
	Processing time (s)	299.8	238.564	181.65	405.97	143.79	195.087	126.229	160.879
LGFI	Iteration	40	250	100	190	50	100	50	50
	Processing time (s)	2.495	12.339	5.275	9.367	2.723	5.582	3.178	3.347
FRAGL	Iteration	30	60	30	50	20	40	20	30
	Processing time (s)	2.095	3.539	1.78	6.669	1.708	3.925	1.173	2.627
HLFRA	Iteration	10	20	20	30	30	30	20	20
	Processing time (s)	1.281	2.231	1.877	2.775	2.652	4.783	1.923	2.663
Proposed SRIS	Iteration	10	15	5	15	15	20	10	15
	Processing time (s)	1.073	1.872	0.803	1.923	1.482	1.519	1.247	1.957

**FIGURE 6.**
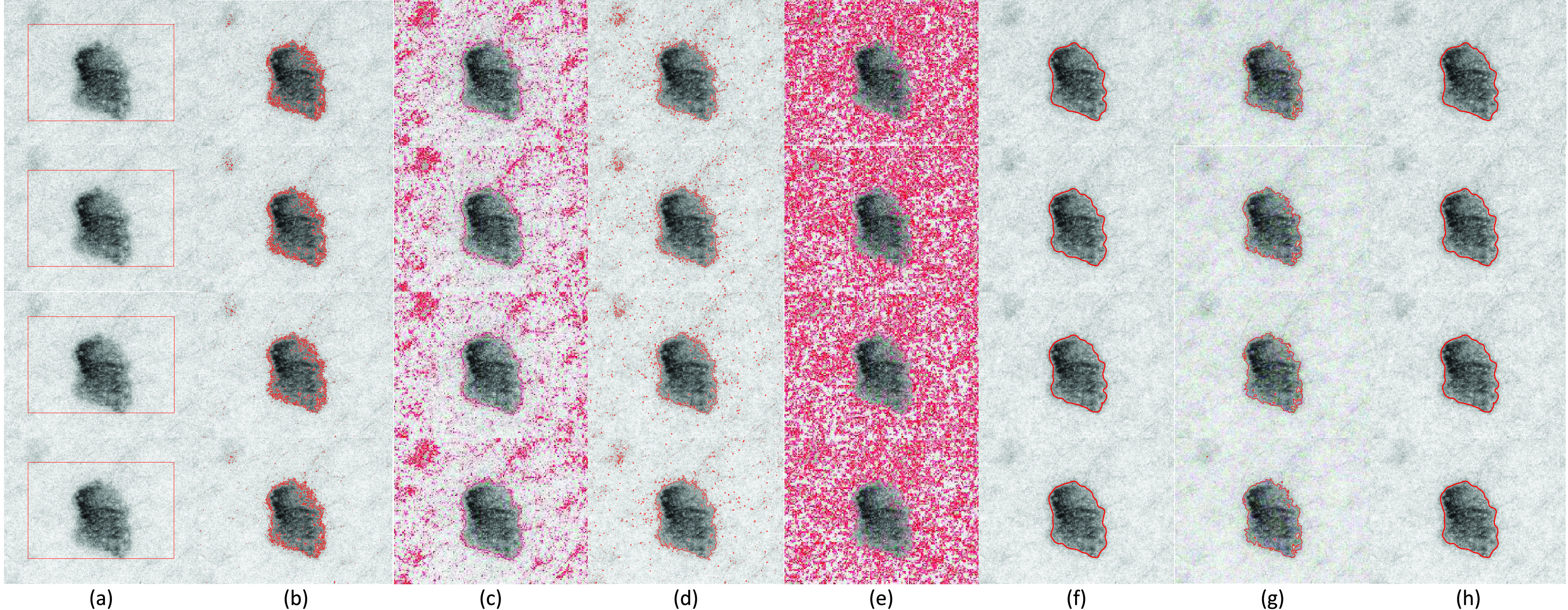
Segmentation results and comparison of the image with varying Gaussian noise level: (0.01, 0.02, 0.03, 0.04). (a) Original image with initial contour, (b) CV, (c) VLSBCS, (d) LSACM, (e) LGFI, (f) FRAGL, (g) HLFRA, and (h) Proposed SRIS.

**FIGURE 7.**
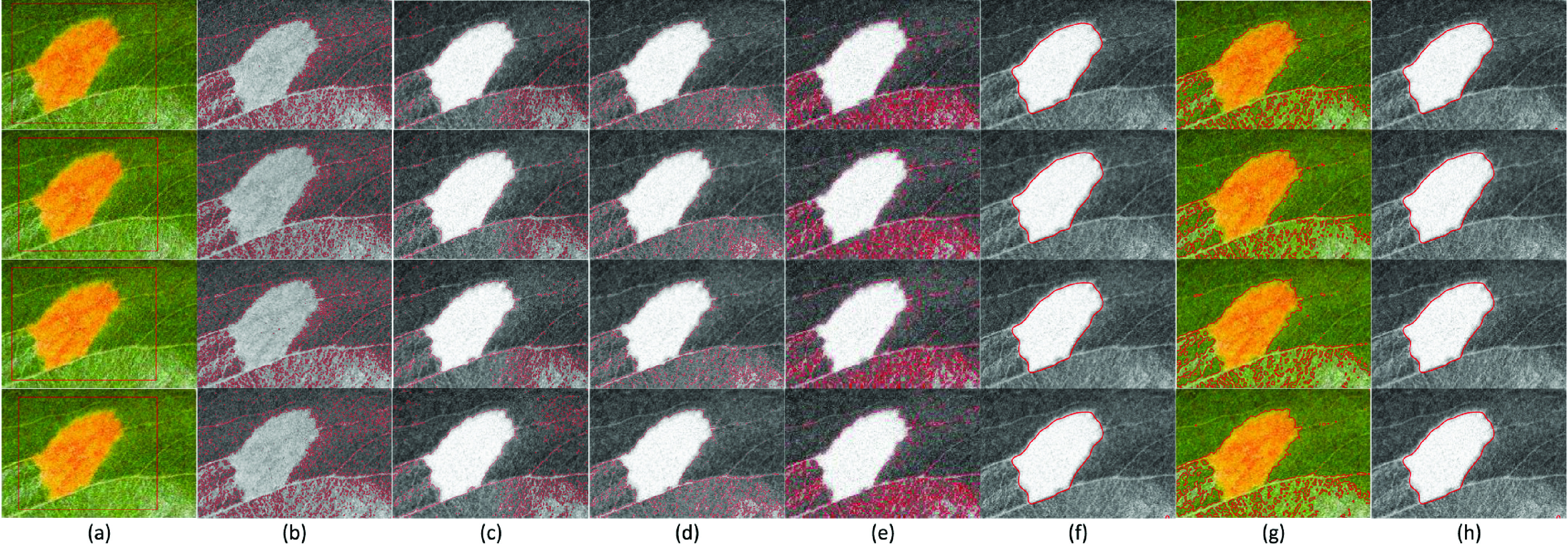
Segmentation results and comparison of the image with varying salt-and-pepper noise level: (0.01, 0.02, 0.03, 0.04). (a) Original image with initial contour, (b) CV, (c) VLSBCS, (d) LSACM, (e) LGFI, (f) FRAGL, (g) HLFRA, and (h) Proposed SRIS.

In Fig. 6 and Fig. 7, two images (one with Gaussian noise and other with salt-and-pepper noise) are used to show the robustness of the proposed SRIS model toward the noise. From Row 1 to Row 4 in Fig. 6 and Fig. 7, noise levels were altered to 0.01, 0.02, 0.03, and 0.04, respectively. The CV, VLSBCS, LSACM, and LGFI models cannot eliminate the noise in the images and generated false contours around the ROI boundaries (Fig. 6,(b)-(e) and Fig. 7,(b)-(e)). Moreover, the FRAGL and HLFRA models achieved excellent performance, making its segmentation accuracy closer to the proposed SRIS model (Fig. 6 (f)-(g) and Fig. 7 (f)-(g)). In the proposed SRIS model, saliency information is used to extract the ROI from the background, and most of the noise is eliminated using the regularization term. The ROI is accurately segmented regardless of the image complexity and noise type; thus, SRIS is robust to noise (Fig. 6(h) and Fig. 7(h)).

**FIGURE 8.**
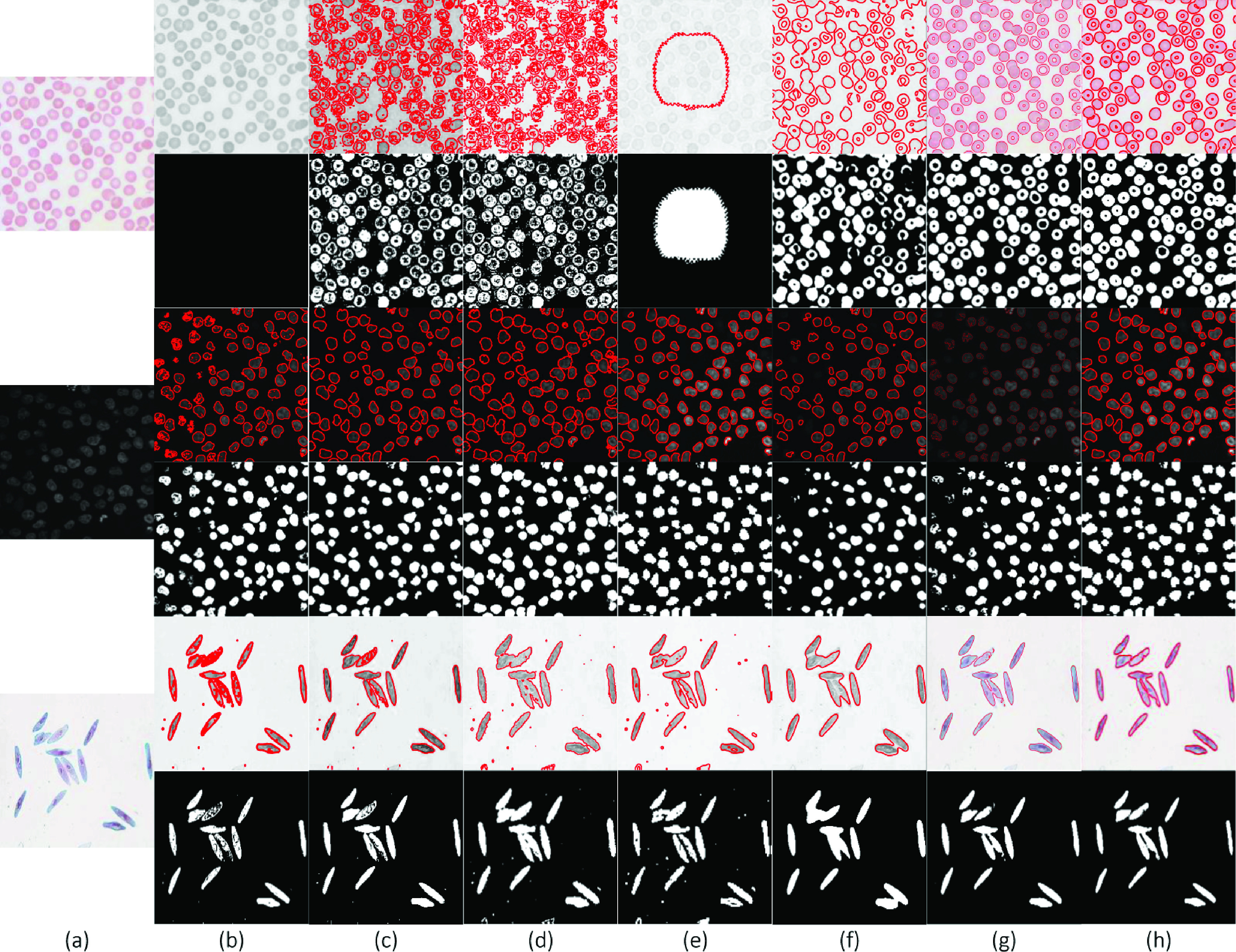
Segmentation results and comparison of cell images. (a) Original images, (b) CV, (c) VLSBCS, (d) LSACM, (e) LGFI, (f) FRAGL, (g) HLFRA, and (h) Proposed SRIS.

Moreover, the state-of-the-art and proposed SRIS models have been evaluated on erythrocyte (first), leukocyte (second), and paramecium images (third), all in Fig. 8(a). The results are shown in Fig. 8,(b)-(h). The CV (Fig. 8(b)) and LGFI (Fig. 8(e)) models cannot segment the first image because the intensities of certain cells are the same as the background, and also the edges of the cells are weak, resulting in leakage. For the other two images, most cells were detected, but the edges were not accurately positioned. Similar results were obtained for VLSBCS, LSACM, FRAGL, HLFRA models, in which some isolated cells were segmented in adhesion, as shown in Fig. 8 (c), (d), (f), and (g), respectively. The proposed model showed less cell adhesion and provided precise segmentation, as shown in Fig. 8(h).

## V. Qualitative and Quantitative Analysis

In this section, the qualitative and quantitative analyses of the proposed SRIS model have been illustrated using COVID-CT [40] and THUS10000 [41] datasets comprising 349 CT images of 216 COVID-19 patients and 10000 real images, respectively. In addition, the segmentation results achieved by the proposed SRIS model are compared with the state-of-the-art models mentioned in Section II.

**FIGURE 9.**
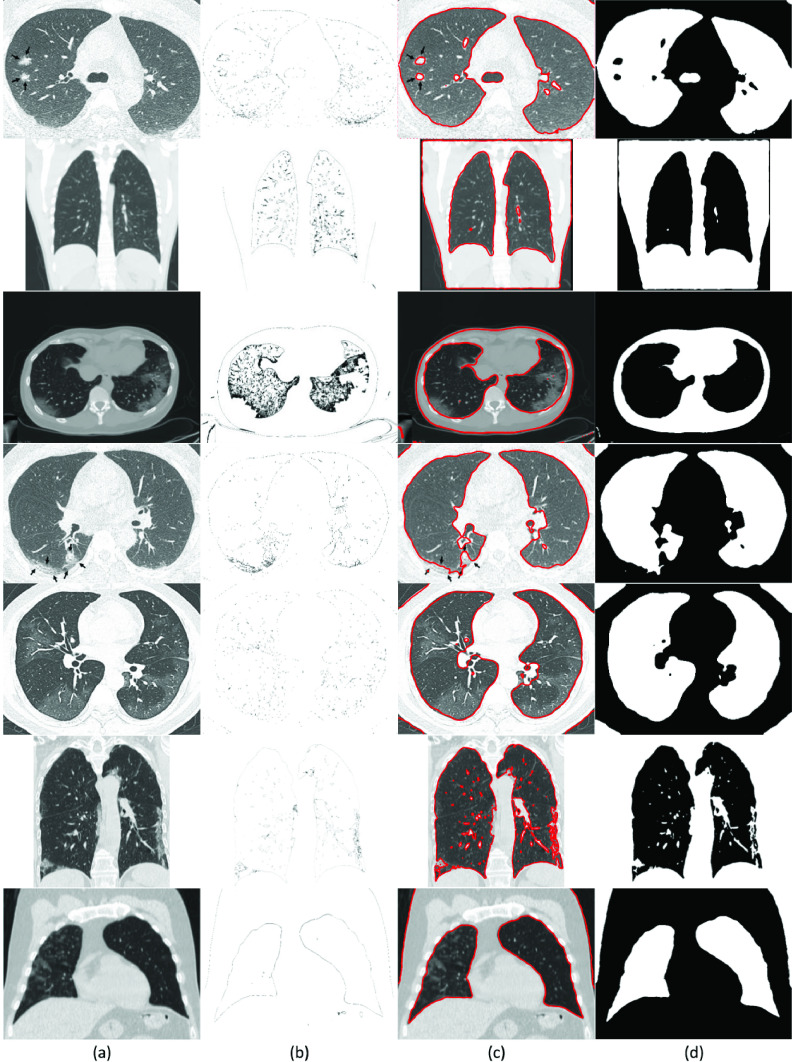
SRIS segmentation results of COVID-19 patients’ lung images. (a) Original image, (b) saliency information, (c) final contour, and (d) segmentation results.

**FIGURE 10.**
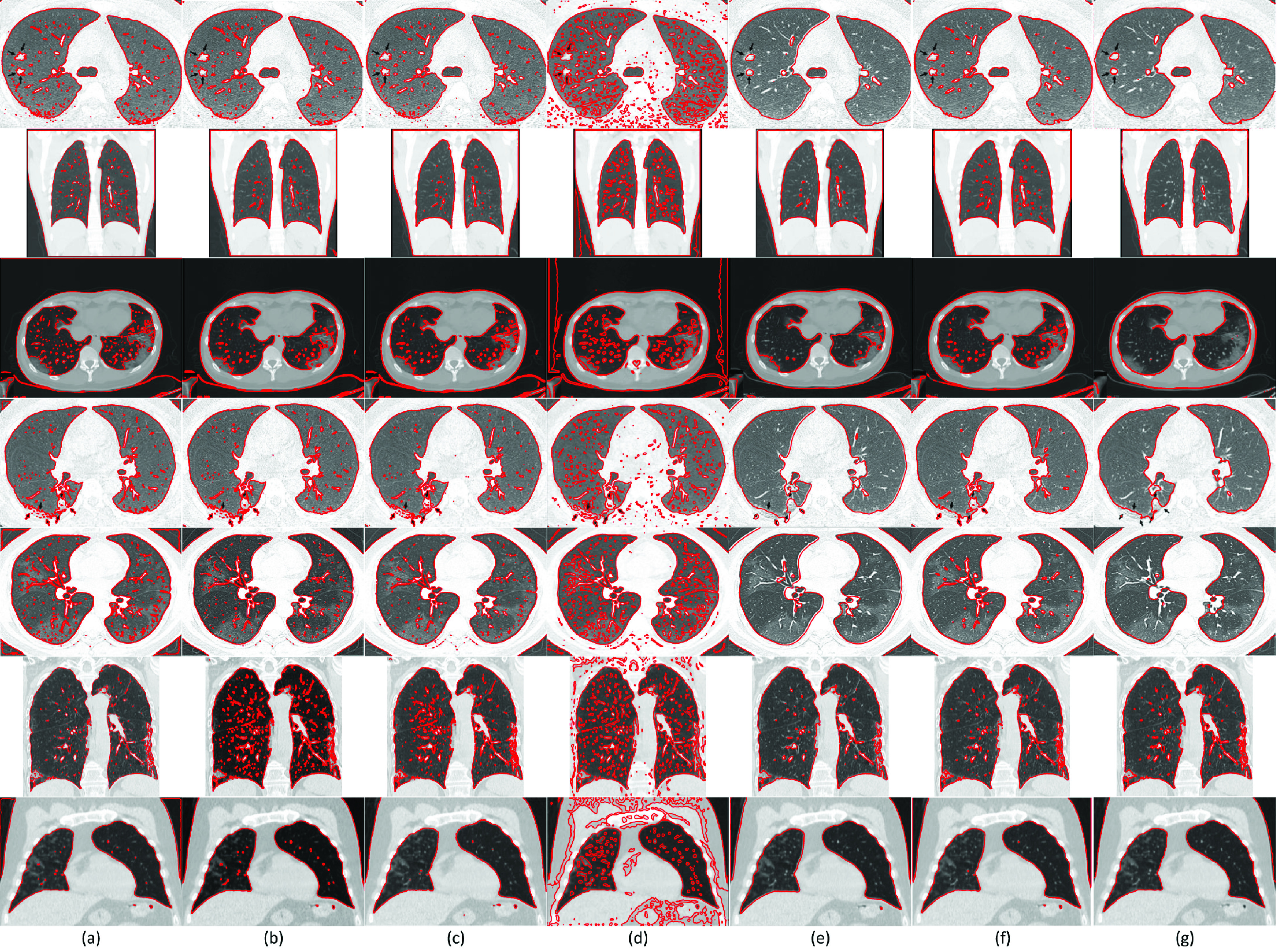
Segmentation results and comparison of COVID-19 patients’ seven lung images from COVID-CT dataset [40]. (a) CV, (b) VLSBCS, (c) LSACM, (d) LGFI, (e) FRAGL, (f) HLFRA, and (g) Proposed SRIS.

To test the reliability of the proposed SRIS model on medical images, CT images from COVID-CT dataset [40] were tested for the segmentation of COVID-19 infected lungs. As an example, Fig. 9 shows the segmentation of seven CT images. In Fig. 9(a), it can be seen that most lesions are located around, with a slight preponderance of dorsal lung regions. Due to the special structure and visual characteristics, it is difficult to distinguish the infected region (shown by arrows in the first and fourth images) boundaries from the chest wall. Thus, the past segmentation models failed to segment the COVID-19 infected area accurately. However, with the incorporation of saliency information in the proposed model, accurate segmentation of the infected areas was obtained. Hence, SRIS is a promising approach for the early screening of COVID-19. According to the lung saliency information in Fig. 9(b), the proposed SRIS model can implicitly find the edges of the lungs for the segmentation, as shown in Fig. 9(c). Therefore, the proposed SRIS model perfectly extracted the lungs from the contrast and the challenging backgrounds, and the results looked closer to manual segmentation. The comparative results with the state-of-the-art models are shown in Fig. 10. It shows that compared with all the state-of-the-art models, the proposed SRIS model provided the best segmentation results for most images.

To analyze the proposed SRIS model quantitatively, the following metrics were calculated: Accuracy, Dice coefficient (DSC), Sensitivity, and Specificity. The results are shown in Table 4. Accuracy metric is the correlation between the segmented and actual regions, DSC metric measures the overlap between the segmented and actual regions, Sensitivity metric characterizes the detected ROI by the segmentation model, and Specificity metric characterizes the region ignored by the segmentation model. These metrics are formulated as follows:

\begin{align*} \text {Accuracy}=&\frac {\text {TP $+$TN}}{\text {TP $+$TN $+$FP $+$FN}}, \tag{34}\\ \text {DSC}=&\frac {\text {2TP}}{\text {2TP $+$FP $+$FN}}, \tag{35}\\ \text {Sensitivity}=&\frac {\text {TP}}{\text {TP $+$FN}}, \tag{36}\\ \text {Specificity}=&\frac {\text {TN}}{\text {TN $+$FP}}. \tag{37}\end{align*}

If the values are close to 1, the results obtained are considered acceptable. Here, TP (true positive) and TN (true negative) represent the correctly segmented and unsegmented regions. FP (false positive) and FN (false negative) represent detected and undetected false-regions, respectively. Table 4 shows that the proposed SRIS model has achieved better values of Accuracy, DCS, Sensitivity, and Specificity compared with the past models.

**TABLE 4:** Average Metric Analysis of COVID-CT Dataset [40]

Model	Accuracy	DSC	Sensitivity	Specificity
CV	0.62	0.5	0.71	0.69
VLSBCS	0.82	0.81	0.79	0.85
LSACM	0.63	0.45	0.69	0.67
LGFI	0.82	0.81	0.83	0.84
FRAGL	0.8	0.74	0.75	0.71
HLFRA	0.78	0.72	0.73	0.70
Proposed SRIS	0.98	0.98	0.97	0.98

**FIGURE 11.**
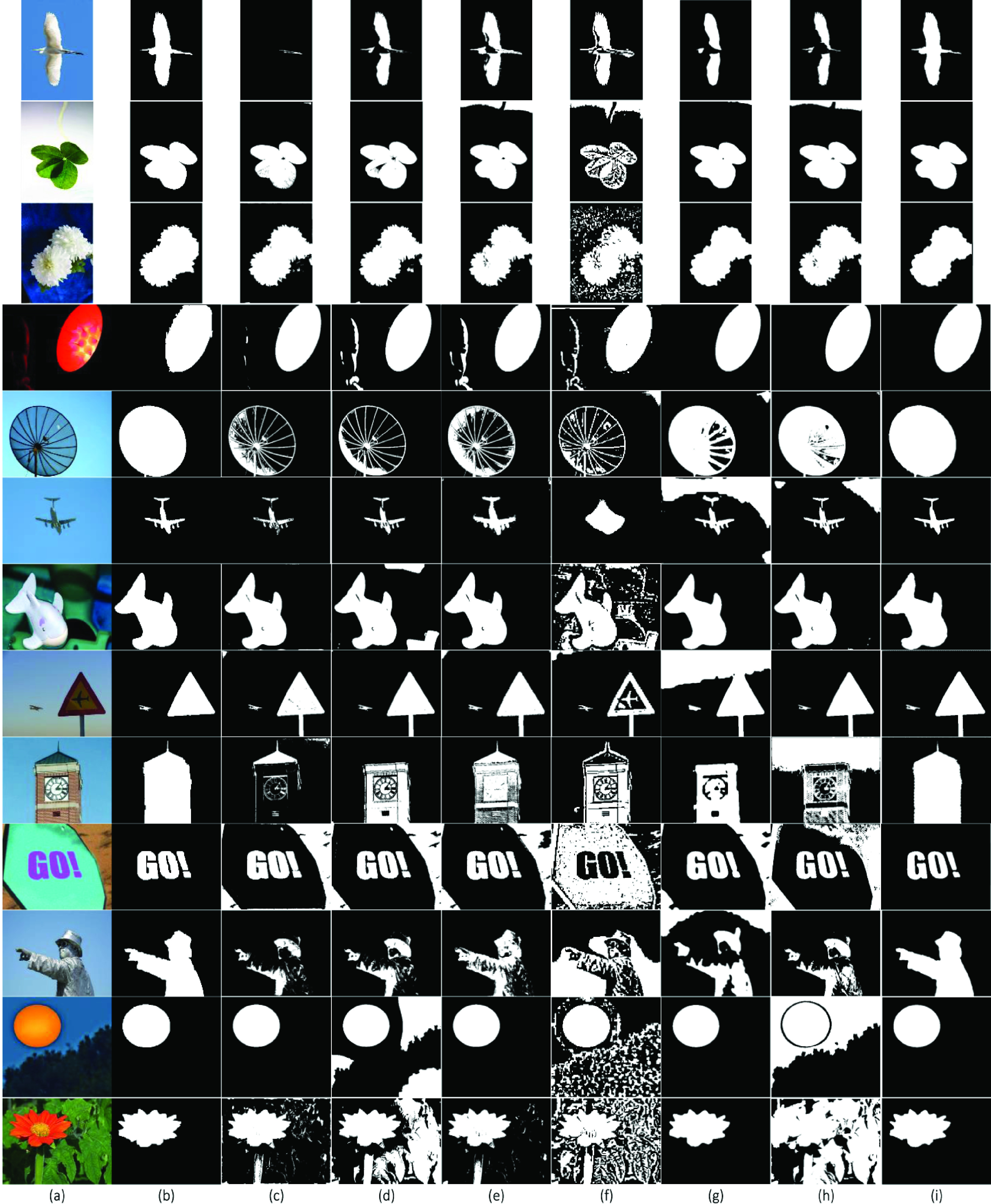
Segmentation results and comparison of 13 real images from THUS10000 dataset [41]. (a) Original images, (b) GT, (c) CV, (d) VLSBCS, (e) LSACM, (f) LGFI, (g) FRAGL, (h) HLFRA, and (i) Proposed SRIS.

In addition, THUS10000 dataset [41], which included 10000 real images, was used to test the accuracy and processing time of the proposed SRIS model in the context of qualitative and quantitative analysis. The segmentation of the SRIS model on 13 images from the dataset, along with the results of past models, are shown in Fig. 11. As illustrated in Fig. 11, in most cases, the proposed SRIS model yielded the best segmentation results.

**FIGURE 12.**
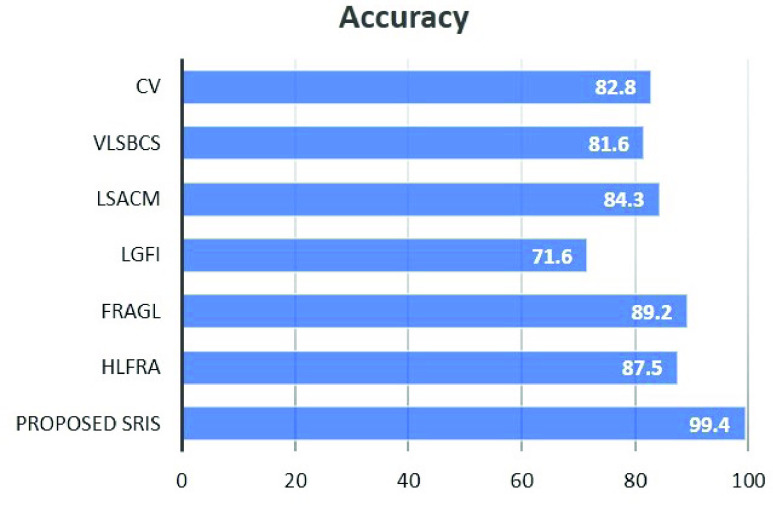
Average accuracy comparison for THUS10000 dataset.

**FIGURE 13.**
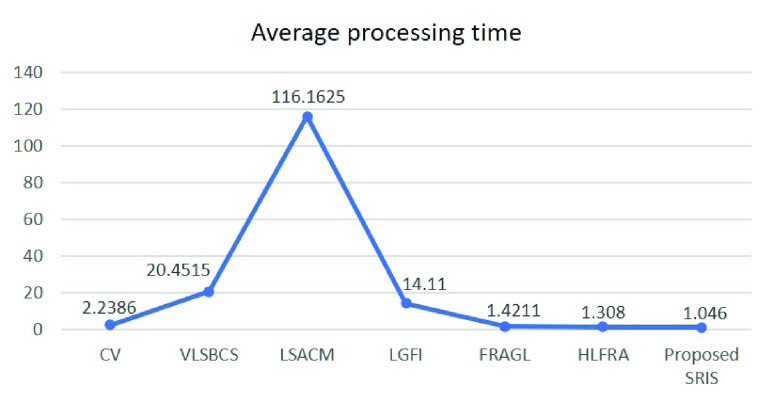
Average processing time for THUS10000 dataset.

Fig. 12 and Fig. 13 show the average accuracy and average processing time required for the segmentation of 10000 real images from THUS10000 dataset [41] using the proposed SRIS model and the state-of-the-art models, respectively. Here, the segmentation accuracy is obtained by comparing the segmented region $R_{S}$ obtained by the segmentation models and the image’s given ground-truth $R_{G}$ as

\begin{equation*} \text {Accuracy} = \frac {{\left |{ {R_{S} \cap {R_{G}}} }\right |}}{{\left |{ {R_{S} \cup {R_{G}}} }\right |}}\%. \tag{38}\end{equation*}

The analyses in Fig. 12 and Fig. 13 show that the proposed SRIS model yielded the highest accuracy with significantly lower processing time.

## VI. Conclusion

In this study, a novel SRIS model was proposed to overcome the problem of image segmentation in the presence of intensity inhomogeneity and noise. A new adaptive level-set evolution protocol based on internal and external functions was designed. The proposed approach eliminated the need for contour initialization and made the proposed model robust to initialization. In the level-set energy function, an adaptive weight function was formulated to adaptively alter the intensities of internal and external energy functions according to the image information. Further, sign of the energy function was modulated depending on the internal and external regions to eliminate the effect of noise in an image. The performance of the SRIS model has been evaluated on complex real and synthetic images with various intensity variations. In addition, the SRIS model was compared with various state of-the-art ACMs in terms of the number of iterations and processing time. The simulation results showed that the proposed SRIS model yielded the best visual segmentation on synthetic and real images. The processing time has significantly reduced compared with the state-of-the-art models. Besides, Accuracy, DCS, Sensitivity, and Specificity metrics were measured for qualitative and quantitative analyses over COVID-CT and THUS10000 real image datasets. According to the results, the proposed SRIS model outperformed all the state-of-the-art models in terms of comparison metrics as well as processing time.
